# Generative AI in Veterinary Pathology: Feasibility of a GPT-Based Assistive Tool for Gross, Cytologic, and Histopathologic Assessment of Canine Cutaneous Neoplasms—A Pilot Study

**DOI:** 10.3390/ani16132070

**Published:** 2026-07-04

**Authors:** Evaristo Di Napoli, Luigi Emiliano Maria Zumbo, Davide De Biase, Giuseppe Piegari, Serenella Papparella, Valeria Russo, Orlando Paciello

**Affiliations:** 1 Department of Veterinary Medicine and Animal Production, University of Naples Federico II, 80137 Naples, Italy; papparel@unina.it (S.P.); valeria.russo@unina.it (V.R.); paciello@unina.it (O.P.); 2 Department of Earth and Marine Sciences (DiSTeM), University of Palermo, 90123 Palermo, Italy; luigiemilianomaria.zumbo@unipa.it; 3 Department of Pharmacy, University of Salerno, 84084 Fisciano, Italy; ddebiase@unisa.it; 4 Department of Human Sciences, Link Campus University, 00165 Roma, Italy; g.piegari@unilink.it; 5 Istituto Zooprofilattico Sperimentale del Mezzogiorno-Portici, 80055 Portici, Italy

**Keywords:** computational dermatopathology, morphological assessment, generative artificial intelligence, decision support systems, image-based pathology

## Abstract

Canine cutaneous neoplasms are common and morphologically heterogeneous lesions whose diagnosis relies on integrating gross examination, cytology, and histopathology. This retrospective pilot study assessed the feasibility of a multimodal GPT-based large language model as an assistive, not autonomous, tool for standardized description, differential diagnosis generation, and classification support across this diagnostic workflow. Fifty-one histologically confirmed canine cutaneous tumors were retrospectively selected from the laboratory information system of the Veterinary Pathology Laboratory, University of Naples Federico II. For each case, de-identified gross photographs, digitized cytology, and representative histologic images were provided to the model using templated prompts. Model outputs were independently reviewed by two veterinary pathologists, who reached consensus on descriptive quality and diagnostic concordance with the histologic reference diagnosis. Final diagnostic outputs were classified as correct, partially correct, or incorrect. Strict accuracy was defined as the proportion of fully correct diagnoses, whereas broad accuracy combined correct and partially correct outputs considered diagnostically informative. Overall, the model achieved a strict diagnostic accuracy of 66.7% (34/51; 95% CI: 53.0–78.0) and a broad diagnostic accuracy of 90.2% (46/51; 95% CI: 79.0–95.7). Performance was highest in epithelial tumors and lower in mesenchymal and melanocytic tumors, in which the model more often identified broader diagnostic categories than specific histotypes. These findings suggest that GPT-based systems may support report standardization, descriptive consistency, and morphology-driven reasoning in veterinary pathology. However, reduced entity-level specificity, variable descriptive quality, and the risk of plausible but non-concordant outputs require strict human supervision and further validation before routine implementation.

## Simple Summary

Generative Pre-trained Transformer (GPT) is an artificial intelligence model trained on large textual corpora and able to generate coherent text and support diagnostic reasoning. This study explored its potential application in veterinary oncology, focusing on skin tumors, among the most frequent neoplasms in dogs. GPT was tested at different stages of the diagnostic workflow—macroscopic description, cytological evaluation, and histological interpretation—using digital images. The model generated standardized descriptive reports, suggested differential diagnoses consistent with morphologic features, and recalled reference classifications such as the World Health Organization (WHO). Results indicate that GPT may serve as an educational and operational aid, promoting language standardization and diagnostic consistency. However, limitations remain, including a lack of clinical validation, risk of misleading outputs, and the need for specialist supervision. With appropriate regulation and integration into digital pathology, GPT could support pathologists in improving the standardization, traceability, and organization of veterinary oncological diagnoses.

## 1. Introduction

### 1.1. Artificial Intelligence: Diagnostic Innovation in Veterinary Oncology

Neoplastic diseases represent a major challenge in veterinary medicine due to marked biological heterogeneity, genomic instability, variable metastatic potential, and the limited availability of tumor-specific therapeutic options. Their clinical and pathological management is further complicated by the broad spectrum of biologic behavior displayed by different tumor types, ranging from slowly progressive lesions to highly aggressive malignancies characterized by local invasion, recurrence, and distant metastasis [1,2,3]. Therefore, accurate diagnostic characterization is essential for prognostic assessment, therapeutic planning, and clinical decision-making.

Among veterinary neoplasms, canine cutaneous tumors are of particular relevance because of their high prevalence in routine diagnostic practice and their marked morphologic diversity. Their evaluation is traditionally based on the integration of gross examination, cytologic assessment, and histopathologic interpretation within internationally recognized classification systems, including those established by the World Health Organization (WHO) and coded according to the canine International Classification of Diseases for Oncology (Vet-ICD-O-Canine-1, a System for Coding Canine Neoplasms Based on the Human ICD-O-3.2) [1,2,3,4,5]. However, the interpretation of these lesions may be challenging because of overlapping morphologic patterns, variable degrees of differentiation, and the need to correlate microscopic findings with biologic and clinical behavior.

In recent years, artificial intelligence (AI) has gained increasing attention in veterinary oncology and diagnostic pathology, particularly in the context of digital pathology. AI-based approaches may enhance diagnostic workflows by improving pattern recognition, increasing reproducibility, facilitating data processing, and promoting greater standardization in pathologic evaluation [1,2,3,4,5]. Although most current developments have focused on image-based systems and deep learning applications, Large Language Models (LLMs) are emerging as complementary tools in diagnostic practice. Models such as Generative Pre-trained Transformer (GPT) may assist in the structuring of pathology reports, the harmonization of diagnostic terminology, and support for clinical and morphologic reasoning, thereby representing a potentially valuable adjunct to computational methods in digital veterinary pathology [6,7]. In parallel, machine learning-based approaches are also being investigated for canine cancer detection and risk stratification, further supporting the expanding role of computational tools in veterinary oncology [8].

AI is rapidly reshaping veterinary diagnostic workflows, particularly in veterinary pathology, by augmenting tasks ranging from slide digitization and image analysis to report standardization and decision support. Whole-slide imaging (WSI) enables end-to-end digital workflows—remote consultation, archiving, and integration of AI models for detection, grading, and quantification, thereby improving scalability and turnaround time while preserving traceability [9,10].

In routine practice, AI adoption is most mature in cytology and clinical pathology: point-of-care digital cytology with embedded deep learning can triage cases, flag atypical cells, and standardize descriptions, complementing specialist review and accelerating case prioritization [11,12,13].

Beyond image-centric deep learning, generative AI—particularly LLMs such as GPT—introduces capabilities for text understanding and synthesis, including drafting standardized macroscopic descriptions, proposing morphology-consistent differential diagnoses, and retrieving relevant classification frameworks. These features support narrative consistency across grossing, cytology, and histopathology while keeping the pathologist “in the loop” [7].

Recent evaluations of LLMs in veterinary domains suggest supportive performance on knowledge and reasoning benchmarks but also highlight brittleness, hallucinations, and the need for guardrails, validation, and governance before clinical deployment [14].

A pragmatic integration pattern is emerging: WSI and digital cytology as the substrate; task-specific deep learning models for detection/segmentation/quantification; LLMs to structure free-text inputs (clinical history, gross descriptions) and to generate draft reports, with final sign-out by a board-certified pathologist. This human-AI collaboration can improve report uniformity, reduce cognitive load, and shorten turnaround times [15].

However, safe translation requires rigorous preclinical testing, quality assurance, dataset curation, reproducibility checks, performance monitoring for data drift, and clear accountability frameworks [16,17].

In veterinary settings, additional challenges include heterogeneous scanners and staining, smaller labeled datasets, and variable case-mix across species and practice types; these factors underscore the importance of transparent validation and domain-specific guidelines [3].

In this context, our work examines GPT-assisted support across the veterinary pathology workflow for canine cutaneous neoplasms, assessing contributions to descriptive standardization, differential diagnosis generation, and classification retrieval while explicitly considering limitations and governance requirements.

### 1.2. Large Language Models and ChatGPT in Veterinary Pathology Diagnostics

Recent advances in AI have led to the development of LLMs, systems capable of understanding, generating, and transforming complex texts with an unprecedented level of coherence and depth, comparable to human language. Current applications of AI in veterinary medicine cover a wide range of fields, including automated interpretation of dental radiographs, early detection of colic, and mitotic count assessment in digital pathology [7,18]. In this context, machine learning (ML) enables systems to learn from data without explicit programming, while generative AI (genAI) represents the subset of ML specifically focused on the creation of new content.

Within genAI, GPT models currently represent one of the most advanced tools in the field of natural language processing, owing to the transformer architecture and the emergent capabilities developed through training on large-scale textual datasets. The evolution from GPT-3 to multimodal models such as GPT-4 and GPT-4o has highlighted how large neural networks can develop emergent abilities in clinical reasoning, problem solving, and structured information synthesis, even in highly specialized domains such as human and veterinary medicine [7].

In recent years, the integration of generative AI into veterinary sciences has increased rapidly, with studies demonstrating that LLMs possess competencies comparable to those of advanced veterinary students or medical trainees in tasks involving clinical reasoning and interpretation of medical history [14]. These models offer new opportunities in the drafting of clinical notes, interpretation of anamnesis data, triage support, and organization of clinical information into coherent and standardized formats [7].

At the same time, the growing diffusion of digital pathology and WSI has created an ideal context for integrating GPT models into the diagnostic workflow. Digitized slides, deep learning pipelines, and automated classification systems provide the infrastructure that enables language models to translate histological, cytological, and macroscopic patterns into coherent diagnostic descriptions [11].

The possibility of using GPT to generate preliminary diagnostic reports, extract key information, formulate plausible differential diagnoses, and retrieve authoritative classification systems (such as WHO classifications or established histological schemes) paves the way for a new form of human–AI collaboration, in which the pathologist guides, supervises, and validates the model’s output [7].

At the same time, the recent literature highlights the limitations of LLMs—such as the potential generation of incorrect information (hallucinations), diagnostic bias, or misleading justifications—and emphasizes the need to integrate these tools exclusively within a human-in-the-loop framework, with final supervision and responsibility remaining with the pathologist [14,15,16].

Overall, the use of LLMs such as GPT represents one of the most potentially useful innovations in the modernization of veterinary diagnostics, particularly in a context where digital pathology, point-of-care digital cytology, and deep learning pipelines already constitute the foundation of the diagnostic workflow [11].

### 1.3. GPT Model Architecture and Principles of Operation

GPT models are based on transformer architecture and are designed to process and generate structured textual information. With the introduction of multimodal capabilities, recent GPT-based systems can integrate text and images, allowing their application to workflows that include reports, clinical information, and selected morphologic images. Although these systems are not specifically optimized for histopathological image feature extraction, they may support the organization of morphologic observations, the generation of structured descriptions, and the formulation of morphology-consistent diagnostic hypotheses under pathologist supervision [7,14].

### 1.4. Canine Tumors of the Skin: Diagnostic Workflow from Gross to Histology

Cutaneous neoplasms are among the most commonly diagnosed tumors in dogs, encompassing epithelial, mesenchymal, round-cell, and melanocytic lineages with heterogeneous biological behavior. Risk is partly modulated by environmental exposures, opening avenues for comparative oncology; moreover, because companion animals share human environments, they serve as sentinel species for environmental carcinogenic exposures [19].

Diagnostic accuracy relies on a stepwise workflow integrating gross examination, cytology, and histopathology, each contributing complementary evidence to case stratification and clinical decision-making. Authoritative compendia (e.g., Tumors in Domestic Animals; Goldschmidt & Hendrick’s skin/soft-tissue chapter) emphasize standardized lesion description, sampling, and correlation with clinical context as prerequisites to robust diagnosis and prognosis [20,21].

At the macroscopic level, systematic recording of location, dimensions, growth pattern, borders, ulceration, and fixation to adjacent structures guides differential diagnosis and selection of margins for excision or biopsy. Consistent grossing templates also facilitate downstream correlation with cytologic and histologic findings [21].

Fine-needle aspiration (FNA) cytology is widely adopted as a minimally invasive first-line test that can rapidly triage common entities—round-cell tumors (e.g., mast cell tumor, histiocytoma, lymphoma, plasma cell tumor), epithelial neoplasms, and soft-tissue sarcomas—while informing sampling strategy and client communication. Pattern-based cytologic interpretation (cellularity, cell cohesion, nuclear criteria, background) underpins high diagnostic yield when integrated with lesion history and gross features [22].

Definitive classification and grading depend on histopathology, ideally on well-oriented, adequately margined samples with ancillary stains as needed. For canine mast cell tumor (a prototypical and prevalent cutaneous neoplasm), histologic grading conveys powerful prognostic information. The Kiupel two-tier system improves interobserver agreement and simplifies risk stratification compared with the historical Patnaik three-tier scheme, although both remain in use and should be interpreted alongside mitotic index, margins, and clinical variables [23,24,25,26].

Consensus guidance further recommends transparent reporting, explicit grading criteria, and alignment of pathology readouts with therapeutic decisions [27].

In sum, a coherent gross-cytology-histology pipeline—anchored in standardized descriptors, appropriate sampling, and validated grading systems—remains central to accurate diagnosis and prognostication of canine cutaneous neoplasms and provides the framework within which emerging tools (e.g., digital pathology and AI) can be safely layered [20].

### 1.5. AI Across Macro and Micro for Canine Cutaneous Tumors

AI is increasingly being investigated across the diagnostic continuum of canine skin neoplasms, from macroscopic imaging to cytology and histopathology. While image-based deep learning approaches are more directly suited to detection, classification, and grading tasks, GPT-based models may provide complementary support by structuring descriptive information and harmonizing diagnostic language. Such integration should remain within a human-in-the-loop framework, with final interpretation and responsibility maintained by the veterinary pathologist [3,7,10,11,28,29].

### 1.6. Study Objectives

This pilot study aimed to assess the feasibility of a multimodal GPT-based LLM as an assistive—not autonomous—tool across the diagnostic workflow of canine cutaneous neoplasms. Specifically, we evaluated its ability to generate standardized gross, cytologic, and histopathologic descriptions, propose morphology-consistent differential diagnoses, and reference relevant classification frameworks. Performance was assessed by expert review of descriptive quality and diagnostic concordance with histopathology as the reference standard.

## 2. Materials and Methods

### 2.1. Case Selection and Data Set

We retrospectively identified 51 canine cutaneous neoplasm cases from the My Clinical/My Anatomy laboratory information system of the Veterinary Pathology Laboratory, Department of Veterinary Medicine, University of Naples Federico II.

Cases were included when a final histologic diagnosis was available and when the corresponding record contained sufficient material for the multimodal workflow, including clinical descriptors, gross photographs, cytologic preparations, and scanned histology, from which representative fields were selected. Cases with incomplete documentation, non-diagnostic cytologic or histologic material, or insufficient image quality for model prompting were excluded. Cutaneous tumors were chosen because they are directly approachable at external examination and amenable to a stepwise diagnostic workflow (gross → cytology → histology).

For every case, standardized gross images were acquired, and both cytologic smears and histologic sections were prepared according to routine protocols. All materials were de-identified before analysis. A GPT-5-based LLM (OpenAI) was used to process textual information and to analyse images through vision-enabled prompts. We designed task-specific prompts intended to minimize leading language and reduce anchoring bias.

### 2.2. Macroscopic Examination (LLM Tasks)

Macroscopic prompting was conducted in three predefined steps, without allowing the model to infer a diagnosis from non-visual information:

Structured description (no diagnosis): detailed and objective description limited to location, size, shape, color, borders/margins, ulceration, presumed consistency, and relationship to adjacent tissues.

Top 3 differentials: a list of three morphology-based differential diagnoses with assigned probabilities (percent values summing to 100%); no references to cytology or histology were allowed at this stage.

Most likely macroscopic diagnosis: one accurate macroscopic hypothesis with a ≤5-line rationale grounded solely in visible features.

### 2.3. Microscopic Examination: Cytology and Histology (LLM Tasks)

For image input, each case contributed 20 representative fields (10 images at 20× and 10 images at 40×), selected by a pathologist (E.D.N.) to capture diagnostically informative areas and avoid artifacts. Given the large number of image inputs generated across all cases, representative de-identified images used for model prompting are provided as Supplementary Material to illustrate the type and quality of the material analyzed.

#### 2.3.1. Cytology

The model was constrained to the following:

Structured description (no diagnosis): report limited to cellularity, cell population(s), and putative origin, cohesiveness, staining affinity, background elements/haematic contamination, and cytologic atypia (e.g., anisocytosis, anisokaryosis, mitoses), listing populations in decreasing order of abundance.

Top 3 differentials: three cytology-based differential diagnoses with probabilities; at this stage, the model was allowed to incorporate the corresponding macroscopic information.

Most likely cytologic diagnosis: one accurate cytologic hypothesis with a ≤5-line rationale grounded in the observed microscopic features.

#### 2.3.2. Histology

Prompts for histology mirrored the cytology structure but focused on tissue architecture:

Structured description (no diagnosis): identification of tissue type and anatomic relationships, growth pattern (exophytic vs. infiltrative), cellular density, circumscription/capsule, stromal features (type and prominence), description of neoplastic cell populations (origin, relative abundance), and histologic atypia/mitoses.

Top 3 differentials: three histopathology-based differentials with probabilities, explicitly permitted to integrate the paired gross and cytologic information.

Most likely histologic diagnosis: one accurate histologic diagnosis with a ≤5-line rationale grounded in microscopic evidence.

#### 2.3.3. Prompting and Quality Controls

Prompts were templated and reused verbatim across cases to support standardization. The model was instructed to avoid external knowledge beyond the provided images/text and to separate description from interpretation.

Outputs were later evaluated for descriptive coherence/completeness, concordance of differentials with observed morphology, and pertinence/correctness of classification references (WHO) when invoked by the model.

Two veterinary pathologists (E.D.N. and O.P.) independently reviewed each case. For cytology, reviewers assessed the LLM’s descriptive reports and differentials against the cytologic preparations; diagnostic performance was referenced to the corresponding histologic diagnosis (gold standard). For histology, reviewers assessed descriptions, differentials, and the most likely diagnosis directly on the histologic preparations. Reviewers were blinded to the model’s outputs when establishing the reference diagnosis and to each other’s assessments. The model was, in turn, not exposed to reviewer comments. Each output was scored using predefined rubrics: description completeness/coherence, appropriateness of Top 3 differentials, classification framework suitability, and final accurate diagnosis.

The finalized consensus served as the reference standard for all performance summaries and error analyses.

### 2.4. Output Evaluation and Diagnostic Concordance

Each model-generated response was reviewed according to a predefined rubric developed to assess the overall diagnostic quality of the output. The evaluation considered four main domains: completeness and coherence of the morphologic description, appropriateness of the Top 3 differential diagnoses, adequacy of the proposed classification framework, and accuracy of the final diagnosis in comparison with the histologic reference standard. To provide a clinically oriented interpretation of model performance, the final diagnostic output for each case was additionally categorized according to its degree of concordance with the reference diagnosis. Outputs were classified as correct (C) when fully concordant with the histologic gold standard; partially correct (PC) when not completely overlapping but still diagnostically compatible, such as in cases in which the model correctly identified the tumor family, morphologic pattern, or broader diagnostic category without reaching the exact histotype; and incorrect (I) when the proposed diagnosis was considered not compatible with the reference standard.

This evaluation framework was intended to capture both the qualitative organization of the model response and its practical diagnostic relevance in the context of veterinary pathology.

### 2.5. Statistical Analysis

Statistical analysis was performed to provide a descriptive assessment of model diagnostic performance. The analytical dataset included, for each case, the following variables: case identifier, tumor category, histologic gold standard diagnosis, model output, and final diagnostic concordance. Tumors were grouped into four diagnostic categories: epithelial (E), mesenchymal (M), round-cell (R), and melanocytic (Mel). Final diagnostic concordance was classified as correct (C), partially correct (PC), or incorrect (I), according to the degree of agreement between the model-generated diagnosis and the histologic reference standard. Based on this classification, diagnostic performance was summarized using descriptive metrics. Strict accuracy was defined as the proportion of fully correct diagnoses among all evaluated cases. Broad accuracy was defined as the proportion of diagnostically informative outputs, combining correct and partially correct results. The incorrect rate was defined as the proportion of outputs considered not compatible with the reference diagnosis. These metrics were calculated for the overall dataset and separately for each tumor category.

Categorical variables were summarized as absolute frequencies and percentages. All main performance estimates were reported with corresponding 95% confidence intervals (95% CI), calculated using the Wilson score method. Given the exploratory design of the study and the relatively limited sample size, the statistical approach was primarily descriptive and focused on estimation rather than formal hypothesis testing. No formal inferential comparisons were planned a priori. Statistical analyses were performed using R 4.5.1 software (R Foundation for Statistical Computing, Vienna, Austria).

## 3. Results

A total of 51 canine cutaneous neoplasms were included in the study. The dataset comprised 13 epithelial tumors, 17 mesenchymal tumors, 18 round-cell tumors, and 3 melanocytic tumors. The dataset was intended to include a spectrum of routinely encountered canine cutaneous tumors across the main diagnostic categories. However, it was not designed to reproduce the true prevalence of each tumor entity in routine diagnostic caseloads. For each case, the histologic reference diagnosis, model output, and final diagnostic concordance were recorded and included in the analysis. The complete case-level dataset, including tumor category, histologic gold standard diagnosis, model-generated output, and final diagnostic concordance, is reported in Table 1.

Diagnostic concordance was interpreted as a downstream measure of the diagnostic usefulness of the model-generated descriptions, rather than as the study’s sole endpoint. Therefore, in addition to the final diagnostic label, the qualitative content of the outputs was reviewed to assess whether the model could organize relevant morphologic information coherently and in a diagnostically meaningful way.

Overall, the model achieved a fully correct final diagnosis in 34 of 51 cases, corresponding to a strict accuracy of 66.7% (95% CI: 53.0–78.0). When partially correct outputs were also considered diagnostically informative, broad accuracy increased to 90.2% (46/51; 95% CI: 79.0–95.7). Five cases were classified as incorrect, corresponding to an incorrect rate of 9.8% (95% CI: 4.3–21.0).

When stratified by tumor category, epithelial neoplasms showed the highest strict accuracy, with 12 of 13 cases correctly classified (92.3%; 95% CI: 66.7–98.6), followed by round-cell tumors, with 11 of 18 fully correct diagnoses (61.1%; 95% CI: 38.6–79.7), and mesenchymal tumors, with 10 of 17 correct diagnoses (58.8%; 95% CI: 36.0–78.4). Melanocytic tumors showed the lowest strict accuracy, with 1 of 3 cases fully concordant with the histologic reference diagnosis (33.3%; 95% CI: 6.1–79.2).

When partially correct outputs were also considered diagnostically informative, broad accuracy reached 100.0% for epithelial tumors (13/13; 95% CI: 77.2–100.0) and melanocytic tumors (3/3; 95% CI: 43.9–100.0), 88.2% for mesenchymal tumors (15/17; 95% CI: 65.7–96.7), and 83.3% for round-cell tumors (15/18; 95% CI: 60.8–94.2). The distribution of correct, partially correct, and incorrect outputs across tumor categories, together with strict and broad accuracy values, is summarized in Table 2. Importantly, most discordant outputs were categorized as partially correct rather than fully incorrect. This finding suggests that, in several discordant cases, the model output retained value at the level of broad morphologic interpretation. In these cases, the model generally identified the appropriate morphologic spectrum, major diagnostic class, or biologically related neoplastic entity while failing to achieve complete concordance with the histologic reference diagnosis at the level of the specific histotype. This pattern was particularly evident in mesenchymal and melanocytic tumors, in which several outputs remained diagnostically informative despite reduced specificity.

Beyond diagnostic concordance, the descriptive component of the model outputs was assessed qualitatively. Overall, GPT-generated descriptions were generally coherent and structured and, when present in the output, included diagnostically relevant morphologic elements such as tumor architecture, cellular arrangement, epithelial or mesenchymal differentiation, nuclear atypia, mitotic activity, stromal features, necrosis, and inflammatory components. The descriptive performance appeared stronger in lesions characterized by well-defined and recurrent histopathological patterns, in which the model was able to use appropriate and relatively specific diagnostic terminology.

In fully concordant cases, the descriptive output generally supported the final diagnosis by capturing the main morphologic features expected for the corresponding tumor entity. This was particularly evident among epithelial tumors and selected round-cell tumors, for which the model often generated outputs that were not only diagnostically concordant but also morphologically plausible and consistent with the reference diagnosis.

By contrast, in partially correct cases, the generated descriptions were usually informative at a broader morphologic level but lacked sufficient specificity to support the exact histotype. In these cases, the model tended to recognize the general tumor family or biologic category while failing to identify the key discriminating features required for more precise subtyping. This pattern was particularly evident in mesenchymal tumors, where outputs frequently converged on the broader diagnosis of soft tissue sarcoma, and in melanocytic tumors, where melanocytoma was repeatedly classified as melanoma.

In incorrect cases, the descriptive reasoning was less reliable and occasionally directed the model toward a biologically plausible but non-concordant diagnosis. These errors suggest that, although the model can generate structured and credible histopathological descriptions, the descriptive output may become misleading when key architectural or cytological discriminators are subtle, underrepresented, or insufficiently captured by the input material. Therefore, the descriptive performance of the model should be interpreted as supportive rather than definitive, requiring expert pathological validation.

A case-level review of the outputs summarized in Table 1 showed that the model performed best in lesions characterized by relatively well-defined and recurrent diagnostic patterns. These cases were also those in which the model-generated descriptions were more likely to contain entity-relevant terminology and morphologic features consistent with the final diagnostic label. Fully concordant outputs were frequently observed in epithelial neoplasms, including squamous cell carcinoma, sebaceous adenoma, sebaceous epithelioma, papilloma, and tricoblastoma. High concordance was also observed in several round-cell tumors, particularly cutaneous mast cell tumors, cutaneous extramedullary plasmacytomas, and cutaneous lymphoma. Among mesenchymal tumors, correct outputs were most commonly obtained in lesions with more recognizable diagnostic labels, such as lipoma, liposarcoma, cutaneous hemangioma, cutaneous hemangiosarcoma, and cases broadly classified as soft tissue sarcoma.

By contrast, discordant outputs were more often related to reduced diagnostic specificity than to completely unrelated classifications. From a descriptive perspective, these outputs were not entirely uninformative; rather, they reflected incomplete morphologic discrimination, with preservation of the broader diagnostic category but loss of subtype-level precision. This pattern was particularly evident among mesenchymal tumors, in which the model frequently converged on the broader label of soft tissue sarcoma when the histologic reference diagnosis was a more specific entity, such as perivascular wall tumor, schwannoma, or fibrosarcoma. A similar tendency was observed in melanocytic tumors, in which melanocytoma was repeatedly classified as melanoma. Fully incorrect outputs were less frequent and were mainly represented by discordant classifications across clearly distinct diagnostic entities, including confusion between cutaneous histiocytoma and mast cell tumor, mast cell tumor and cutaneous lymphoma, or fibrosarcoma and liposarcoma.

Exploratory comparisons between tumor categories were not performed, and the statistical analysis remained descriptive.

## 4. Discussion

Although GPT-based tools have already been explored in veterinary medicine, their systematic evaluation across an integrated gross–cytology–histopathology workflow for canine cutaneous neoplasms remains limited. Therefore, the novelty of the present study does not lie in the general use of GPT in veterinary medicine, but in the structured assessment of its descriptive, differential diagnostic, and classification-support outputs within a multimodal veterinary pathology workflow.

The present study investigated the performance of a GPT-based LLM as an assistive tool in the diagnostic workflow of canine cutaneous neoplasms, spanning gross examination, cytology, and histopathology. Overall, the model achieved a strict diagnostic accuracy of 66.7%, while broad accuracy increased to 90.2% when partially correct outputs were also considered diagnostically informative. These findings indicate that, although exact concordance with the histologic reference diagnosis was not consistently achieved, the model frequently generated outputs that remained clinically and morphologically meaningful. Importantly, diagnostic accuracy should be interpreted in light of the primary aim of the study, which was not to validate GPT as an autonomous tumor classifier but to explore whether its generated descriptions could support morphology-based diagnostic reasoning.

At its current level of performance, particularly considering the strict diagnostic accuracy of 66.7%, the system cannot be used reliably for practical diagnostic purposes without further training, refinement, and validation. Therefore, the present findings should be interpreted as evidence of preliminary feasibility for supervised support rather than as evidence of readiness for clinical diagnostic implementation.

A key aspect of the present results is the discrepancy between strict and broad accuracy. This discrepancy also provides indirect information on the quality of the descriptive output: broad concordance indicates that the model often captured the general morphologic domain of the lesion, whereas the lower strict accuracy reflects limitations in translating those descriptions into precise histotype-level diagnoses. This distinction is particularly relevant in veterinary pathology, where the practical value of a diagnostic support system does not necessarily depend on exact histotype recognition alone. Even when the model failed to provide a fully concordant final diagnosis, it often correctly identified the major morphologic spectrum, tumor family, or broader diagnostic class. From a practical standpoint, this may still be useful in supporting differential diagnosis formulation, guiding descriptive reasoning, and facilitating structured report drafting. Accordingly, the findings suggest that the most realistic role of this type of system is not that of a stand-alone diagnostic classifier, but rather that of a support tool capable of assisting the pathologist in organizing and contextualizing morphologic information [6,7,14,15,16].

The highest performance was observed in epithelial tumors, which showed both high strict accuracy and complete broad accuracy. This may reflect the fact that many epithelial cutaneous neoplasms are characterized by relatively reproducible architectural patterns and more stable descriptive terminology. Entities such as squamous cell carcinoma, sebaceous adenoma, sebaceous epithelioma, papilloma, and tricoblastoma appear to be particularly well represented by language-based diagnostic reasoning, likely because their defining features are more consistently captured in narrative descriptions across gross, cytologic, and histologic settings [20,21,22]. This supports the interpretation that GPT performs better when the diagnostic entity is associated with stable, frequently repeated, and morphologically distinctive language patterns. In contrast, lower strict accuracy was observed in mesenchymal, round-cell, and melanocytic tumors, indicating that these categories remain more challenging for the model when a high degree of specificity is required. The case-level review further clarifies the nature of these discrepancies. In mesenchymal tumors, the model frequently converged on the broader label of soft tissue sarcoma when the reference diagnosis corresponded to more specific entities such as perivascular wall tumor, schwannoma, or fibrosarcoma. This pattern suggests that the model was often able to recognize the correct general biologic and morphologic domain but had difficulty resolving the final diagnosis at the level of the precise histotype. A similar reduction in specificity was observed in melanocytic tumors, in which melanocytoma was repeatedly classified as melanoma. In this context, the model appeared more reliable in capturing overall neoplastic identity than in distinguishing finer prognostic or biologic subdivisions [21,22,23].

This behavior has important implications. On the one hand, it highlights one of the principal advantages of LLM-based systems in pathology: the ability to synthesize descriptive information into coherent, plausible, and diagnostically oriented outputs. This capability may be valuable in standardizing terminology, structuring pathology reports, and supporting hypothesis generation, particularly in digital pathology workflows where narrative consistency and rapid organization of findings are increasingly relevant [7,9,10,11,12,14,15,16,17]. In addition, the model may be useful in educational settings, where it can help trainees structure morphologic descriptions, explore differential diagnoses, and relate observations from gross pathology, cytology, and histology within a unified interpretive framework [7,14].

On the other hand, the study also underscores the main limitations of this approach. Importantly, this study did not aim to train a new AI model. Rather, it evaluated the feasibility of applying an existing GPT-based multimodal system to a small, curated pilot dataset within a supervised veterinary pathology workflow. One major limitation is reduced entity-level specificity. This limitation is partly related to the use of a general-purpose GPT-based model rather than an image-specific or histopathology-trained model. Unlike convolutional neural networks and other dedicated computer vision approaches, GPT-based systems are not specifically optimized for histopathological image feature extraction and may therefore be less suited to identifying subtle architectural details or poorly represented tumor entities from image-based material [3,10,17,18,29]. In oncologic pathology, broad diagnostic alignment is not always sufficient because prognosis, treatment planning, and clinical decision-making often depend on the exact histotype, biologic grade, expected biologic behavior, and, in selected contexts, the identification of molecular or immunohistochemical features that may support therapeutic decision-making [1,20,22,23,26,30]. A model that identifies a lesion as belonging to the correct broad category but fails to distinguish between related yet clinically different entities may still provide useful support but cannot replace specialist interpretation. Therefore, the system should not be used for detailed, unsupervised classification of canine cutaneous tumors, particularly when subtype-level discrimination, grading, prognostic assessment, or therapeutic decision-making is required.

This is particularly relevant for diagnostically heterogeneous groups such as mesenchymal and melanocytic tumors, in which subtle distinctions may carry substantial prognostic significance [20,21,22].

A second limitation concerns the intrinsic nature of language-based reasoning. Large language models do not interpret lesions through direct biologic understanding but through probabilistic associations between textual patterns and diagnostic labels [6,7,14]. As a consequence, their outputs may be coherent and persuasive even when not fully correct. This creates a potential risk of overconfidence, especially when the proposed diagnosis is biologically plausible but still inaccurate. In the present dataset, this risk was reflected by partially correct classifications that remained informative but lacked the precision required for final sign-out. Therefore, the use of LLMs in diagnostic pathology should always be framed within human supervision, with the pathologist maintaining full responsibility for case interpretation [2,7,14,16].

Another relevant point is that performance in the present study was assessed in a controlled and curated setting. Because of the retrospective design, variability in the quality, completeness, and representativeness of the available case material and records may have partially influenced model performance. Differences in the completeness of clinical descriptors, gross photographs, cytologic preparations, and representative histologic fields could have affected the descriptive content generated by the model and, consequently, the final diagnostic interpretation. The model was prompted using selected representative material and a predefined rubric, which was appropriate for standardization but does not fully reproduce the variability of routine practice. In real-world diagnostic settings, image quality, sampling adequacy, lesion heterogeneity, clinical history, and ancillary test availability may differ considerably and influence interpretation. Therefore, the results should be regarded as exploratory and proof-of-concept rather than directly generalizable to all routine cases [3,9,10,11,12,15,16,17,18].

Cytologic evaluation represents a particular challenge for this type of workflow because cytologic samples are inherently heterogeneous, and their diagnostic value depends strongly on smear quality, cellularity, staining characteristics, background material, and selection of representative fields. In the present study, representative cytologic and histologic fields were selected by a pathologist before model prompting, which improved standardization but also introduced an expert-dependent preprocessing step. This requirement may reduce the immediate practical efficiency of the workflow because the time needed for field selection and material preparation must be considered when evaluating real-world implementation.

Accordingly, the present workflow should not be interpreted as demonstrating immediate time-saving or replacement of expert diagnostic evaluation. Its potential contribution is instead methodological and supportive: it provides a controlled framework to assess how a GPT-based system organizes morphologic information, standardizes descriptive language, generates differential diagnoses, and fails or overgeneralizes across gross, cytologic, and histopathologic inputs. In its current form, the workflow may be more relevant for structured report drafting, educational support, second-read assistance, and standardized documentation than for direct acceleration of routine diagnostic sign-out.

The study also has limitations related to sample composition. Class imbalance may have influenced model behavior, as tumor categories and individual entities were not equally represented in the dataset. This issue is particularly relevant for underrepresented tumor types, for which the model may have had fewer recurring linguistic and morphologic patterns to rely on. Consequently, the lower specificity observed in selected mesenchymal and melanocytic tumors should be interpreted not only as a diagnostic limitation but also as a reflection of uneven class representation and variable terminology frequency across tumor entities. Although the overall number of cases was sufficient for a descriptive pilot analysis, the distribution across categories was uneven, with a very small melanocytic subgroup. This necessarily limits the robustness of subgroup-specific conclusions and requires cautious interpretation of category-level performance. In addition, although model outputs were initially reviewed according to a structured qualitative rubric, the final statistical analysis was based on the three-level concordance system (correct, partially correct, incorrect), which more directly reflects diagnostic utility but does not quantitatively capture all dimensions of descriptive quality. Future studies could integrate both approaches more formally by combining case-level rubric scores with diagnostic concordance metrics [14,15,16].

Despite these limitations, the present findings support the potential utility of GPT-based systems as adjunctive tools in veterinary pathology. Their most realistic potential applications appear to lie in structured description, terminology harmonization, assistance in differential diagnosis, and support for morphologic reasoning across different levels of examination [5,7,8,9,10,12,13,14,15]. Rather than replacing diagnostic expertise, these systems may function as cognitive support instruments within digitally integrated workflows, helping pathologists organize observations and articulate more standardized diagnostic outputs. In this sense, the value of the model lies less in autonomous classification than in its capacity to enhance efficiency, consistency, and interpretive support [2,7,10,15,16].

Future work should compare general-purpose GPT-based systems with models and architectures specifically designed for image analysis, including convolutional neural networks, deep-learning pipelines for whole-slide images, vision-language models, and models pre-trained or fine-tuned on clinical and histopathological datasets. Such approaches may be more appropriate for identifying subtle architectural features, improving subtype-level discrimination, and reducing errors in underrepresented tumor entities. Future refinements should also include tumor category-specific and task-specific prompts, standardized field-selection protocols, and structured output templates designed to reduce overgeneralization and improve histotype-level discrimination. Future studies should also apply the same standardized methodology to other generative AI systems to determine whether the observed performance reflects limitations of the specific GPT-based model used here or more general limitations of current multimodal generative AI tools in veterinary pathology. In this perspective, GPT-based models may be most useful when integrated with image-specific artificial intelligence tools, combining structured language generation with dedicated visual feature extraction [3,10,15,17,18,29].

Overall, the data suggest that GPT-based assistance may be particularly useful when integrated into a supervised diagnostic framework, where its strengths in language organization and pattern-oriented reasoning can be exploited without overlooking its limitations in specificity and biologic nuance. The future role of such systems in veterinary oncology will likely depend not only on improvements in model performance but also on careful definition of their intended use: not as independent diagnostic authorities, but as tools that complement and extend the work of the veterinary pathologist [1,3,7,10,14,15,16].

## 5. Conclusions

In conclusion, the present retrospective pilot study supports the feasibility of using a GPT-based large language model as an assistive tool within the diagnostic workflow of canine cutaneous neoplasms, encompassing gross examination, cytology, and histopathology. The model showed a meaningful capacity to generate structured and standardized lesion descriptions, formulate morphology-consistent differential diagnoses, and retrieve diagnostically relevant classification references. However, the quality of these descriptions was variable, and descriptive coherence did not always translate into exact diagnostic concordance.

The main value of this approach lies not in autonomous diagnosis but in its ability to support narrative uniformity, organization of morphologic findings, and diagnostic hypothesis generation across different stages of pathologic evaluation. Reduced specificity in selected tumor categories, together with the occurrence of biologically plausible but non-concordant outputs, confirms that GPT-based systems cannot replace specialist judgment. Therefore, their safest and most realistic implementation is as supervised support tools intended to complement, rather than substitute, the veterinary pathologist [2,7,14].

Further validation on larger, prospective, and more diverse datasets is required before routine implementation. Future studies should also include more formal and quantitative assessments of descriptive quality, refinement of prompt design and evaluation frameworks, and direct comparison with image-specific, deep-learning and vision-language models [3,10,15,17,18,29]. Under these conditions, GPT-based systems may become useful components of future veterinary diagnostic workflows, provided that their use remains anchored to expert oversight and pathology-specific clinical judgment [1,3,7,14,15,16].

At its current level of performance, GPT-based assistance should therefore be regarded as a supervised support tool for structured description and diagnostic hypothesis generation, rather than as a system ready for independent clinical diagnostic use.

## Figures and Tables

**Table 1:** Case-level summary of tumor category, histologic diagnosis, model output, and diagnostic concordance.

ID-Cases	Category (R/E/M/Mel)	Location	Gold Standard (Histological Diagnosis)	Output Model (Top-1)	Outcome (C/PC/I)
C01	E	Haired skin, back region	Squamous cell carcinoma	Squamous cell carcinoma	C
C02	E	Haired skin, back region	Sebaceous adenoma	Sebaceous adenoma	C
C03	E	Haired skin, perineal region	Hepatoid glands adenoma	Hepatoid glands adenoma	C
C04	E	Haired skin, Mammary gland region	Adenocarcinoma	Adenocarcinoma	C
C05	R	Haired skin, scrotum region	Cutaneous mast cell tumor (low grade)	Cutaneous mast cell tumor (low grade)	C
C06	R	Haired skin, thoracic region	Cutaneous mast cell tumor (high grade)	Cutaneous mast cell tumor (high grade)	C
C07	R	Haired skin, back region	Dermic Mast cell tumor (Grade II)	Dermic Mast cell tumor (high grade)	PC
C08	R	Haired skin, interdigital space	Dermic Mast cell tumor (Grade II)	Dermic Mast cell tumor (high grade)	PC
C09	R	Haired skin, Mammary gland region	Cutaneous mast cell tumor (high grade)	Cutaneous mast cell tumor (high grade)	C
C10	R	Haired skin, back region	Cutaneous mast cell tumor (high grade)	Cutaneous mast cell tumor (high grade)	C
C11	R	Haired skin, limb region	Cutaneous extramedullary plasmacytoma	Cutaneous extramedullary plasmacytoma	C
C12	R	Haired skin, labial region	Cutaneous extramedullary plasmacytoma	Cutaneous extramedullary plasmacytoma	C
C13	M	Haired skin, limb region	Fibrolipoma	Fibrolipoma	C
C14	E	Haired skin, breast region	Tricoblastoma	Tricoblastoma	C
C15	M	Haired skin, breast region	Lipoma	Lipoma	C
C16	M	Haired skin, breast region	Lipoma	Lipoma	C
C17	M	Haired skin, back region	Cutaneous haemangiosarcoma	Cutaneous haemangiosarcoma	C
C18	M	Haired skin, thoracic region	Perivascular Wall Tumor (PWT)	Soft tissue sarcoma	PC
C19	M	Haired skin, back region	Soft tissue sarcoma	Soft tissue sarcoma	C
C20	M	Haired skin, rump region	Soft tissue sarcoma	Soft tissue sarcoma	C
C21	R	Haired skin, nasal region	Cutaneous histiocytoma	Cutaneous histiocytoma	C
C22	R	Haired skin, back region	Cutaneous lymphoma	Cutaneous lymphoma	C
C23	Mel	Haired skin, labial region	Melanocytoma	Melanoma	PC
C24	R	Haired skin, back region	Cutaneous extramedullary plasmacytoma	Cutaneous extramedullary plasmacytoma	C
C25	R	Haired skin, labial region	Cutaneous extramedullary plasmacytoma	Cutaneous extramedullary plasmacytoma	C
C26	E	Haired skin, back region	Sebaceous epithelioma	Sebaceous epithelioma	C
C27	M	Haired skin, back region	Cutaneous hemangioma	Cutaneous hemangioma	C
C28	E	Haired skin, ear region	Papilloma	Papilloma	C
C29	Mel	Haired skin, head region	Melanoma	Melanoma	C
C30	E	Haired skin, head region	Sebaceous adenoma	Sebaceous adenoma	C
C31	E	Haired skin, back region	Tricoepithelioma	Tricoepithelioma	C
C32	M	Haired skin, limb region	Schwannoma	Soft tissue sarcoma	PC
C33	M	Haired skin, labial region	Fibroma	Follicular tumor	I
C34	M	Haired skin, back region	Perivascular Wall Tumor (PWT)	Soft tissue sarcoma	PC
C35	Mel	Haired skin, labial region	Melanocytoma	Melanoma	PC
C36	M	Haired skin, breast region	Liposarcoma	Liposarcoma	C
C37	E	Haired skin, breast region	Sebaceous epithelioma	Sebaceous epithelioma	C
C38	M	Haired skin, back region	Fibrosarcoma	Soft tissue sarcoma	PC
C39	M	Haired skin, limb region	Perivascular Wall Tumor (PWT)	Soft tissue sarcoma	PC
C40	M	Haired skin, back region	Soft tissue sarcoma	Soft tissue sarcoma	C
C41	M	Haired skin, rump region	Soft tissue sarcoma	Soft tissue sarcoma	C
C42	R	Haired skin, nasal region	Cutaneous histiocytoma	Cutaneous mast cell tumor (low grade)	I
C43	R	Haired skin, back	Cutaneous mast cell tumor (high grade)	Cutaneous mast cell tumor (high grade)	C
C44	R	Haired skin, labial region	Dermic Mast cell tumor (Grade II)	Dermic Mast cell tumor (high grade)	PC
C45	R	Haired skin, limb region	Dermic Mast cell tumor (Grade II)	Dermic Mast cell tumor (high grade)	PC
C46	E	Haired skin, back region	Squamous cell carcinoma	Squamous cell carcinoma	C
C47	E	Haired skin, back region	Papilloma	Papilloma	C
C48	R	Haired skin, back region	Cutaneous mast cell tumor (high grade)	Cutaneous lymphoma	I
C49	E	Haired skin, ear region	Ceruminous gland adenocarcinoma	Adenocarcinoma	PC
C50	R	Haired skin, head region	Cutaneous histiocytoma	Dermic Mast cell tumor (low grade)	I
C51	M	Haired skin, thoracic region	Fibrosarcoma	Liposarcoma	I

**Table 2:** Diagnostic concordance of model outputs across tumor categories.

Tumor Category	Total Cases (*n*)	Correct, *n* (%)	Partially Correct, *n* (%)	Incorrect, *n* (%)	Strict Accuracy % (95% CI)	Broad Accuracy % (95% CI)
Epithelial	13	12 (92.3)	1 (7.7)	0 (0.0)	92.3 (66.7–98.6)	100.0 (77.2–100.0)
Mesenchymal	17	10 (58.8)	5 (29.4)	2 (11.8)	58.8 (36.0–78.4)	88.2 (65.7–96.7)
Round-cell	18	11 (61.1)	4 (22.2)	3 (16.7)	61.1 (38.6–79.7)	83.3 (60.8–94.2)
Melanocytic	3	1 (33.3)	2 (66.7)	0 (0.0)	33.3 (6.1–79.2)	100.0 (43.9–100.0)
Overall	51	34 (66.7)	12 (23.5)	5 (9.8)	66.7 (53.0–78.0)	90.2 (79.0–95.7)

## Data Availability

All relevant data are listed in the manuscript. Representative de-identified images used for model prompting are provided as Supplementary Material.

## Supplementary Materials

The following supporting information can be downloaded at https://www.mdpi.com/article/10.3390/ani16132070/s1; Supplementary Figure S1: Representative gross, cytologic, and histologic images of a mesenchymal neoplasm, corresponding to case C39, perivascular wall tumor (PWT). Supplementary Figure S2: Representative gross, cytologic, and histologic images of a round-cell neoplasm, corresponding to case C08, dermic mast cell tumor (grade II). Supplementary Figure S3: Representative gross, cytologic, and histologic images of an epithelial neoplasm, corresponding to case C04, adenocarcinoma. Each plate includes a gross image, a cytologic field, and a histologic field with a higher-magnification inset, illustrating the type and quality of the image material used for model prompting.
