# Supplementary figures and images for: Generative AI in Veterinary Pathology: Feasibility of a GPT-Based Assistive Tool for Gross, Cytologic, and Histopathologic Assessment of Canine Cutaneous Neoplasms—A Pilot Study
Source: Animals (Basel). 2026 Jul 4;16(13):2070. doi: 10.3390/ani16132070 (PMC13359981; doi:10.3390/ani16132070)

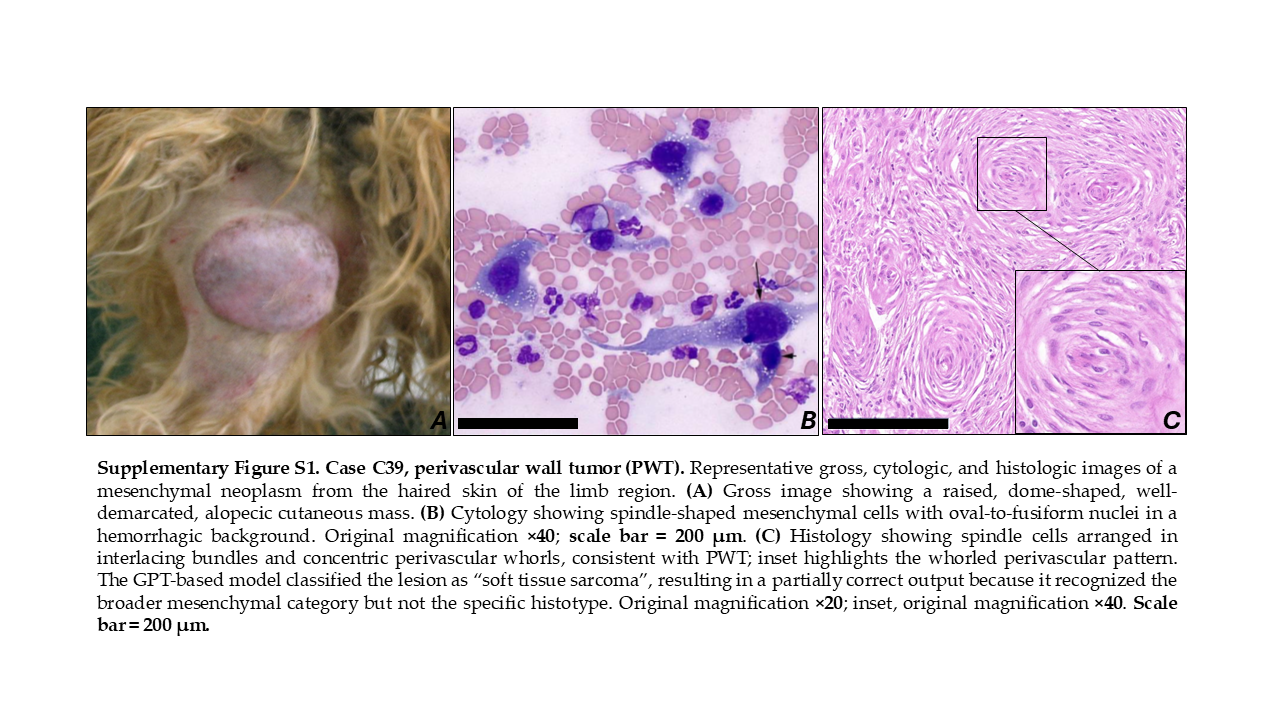

Supplement: Supplementary file 1 [file animals-16-02070-s001.zip › Supplementary Figure S1.tif]

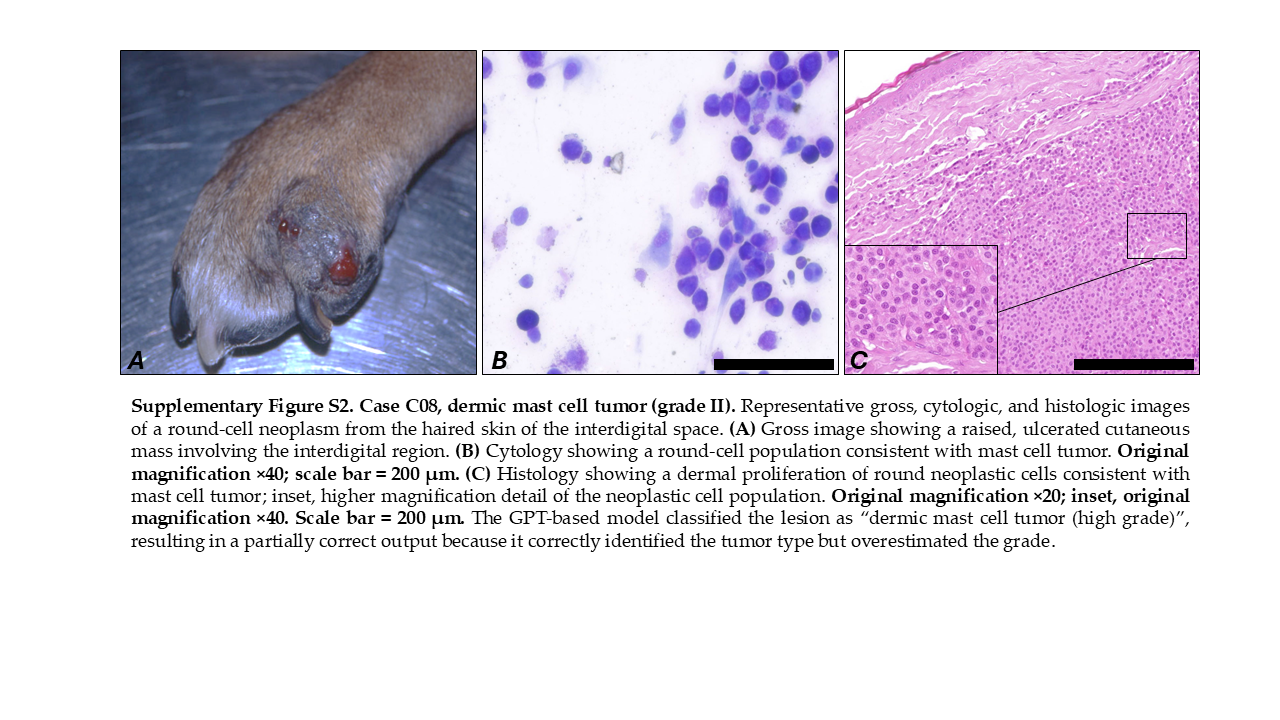

Supplement: Supplementary file 1 [file animals-16-02070-s001.zip › Supplementary Figure S2.tif]

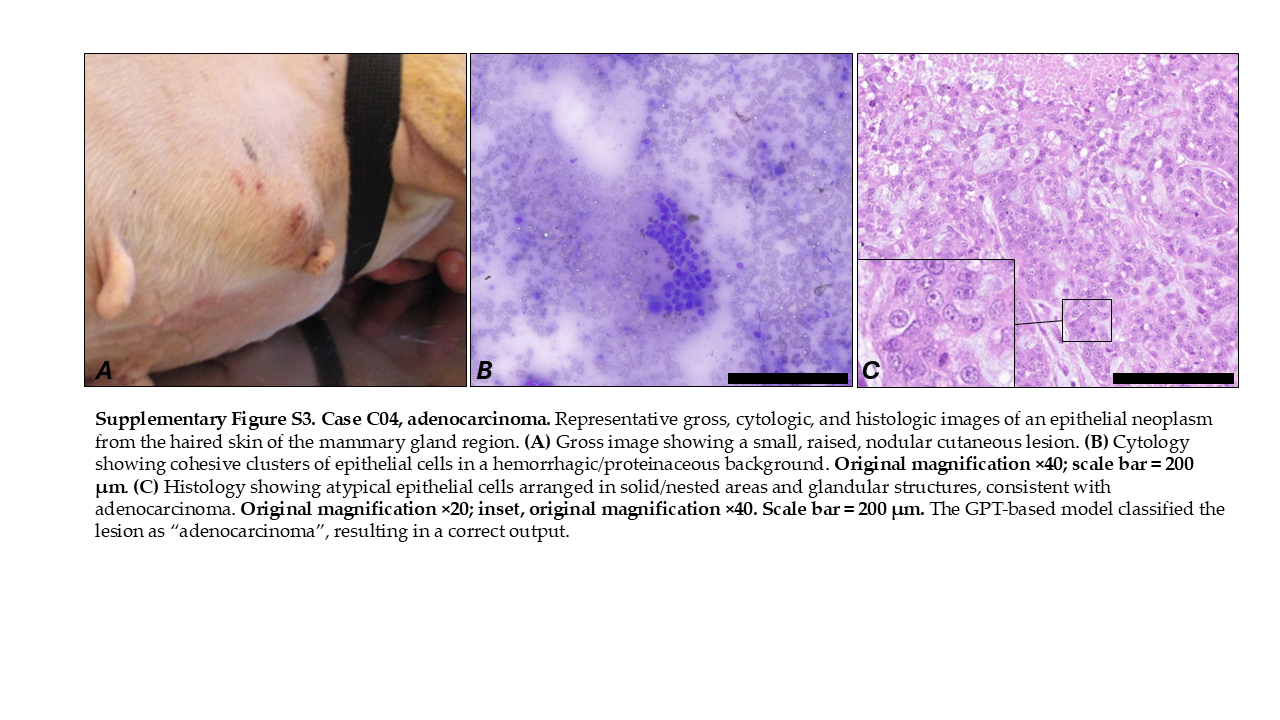

Supplement: Supplementary file 1 [file animals-16-02070-s001.zip › Supplementary Figure S3.tif]
